# Tolerance to FVIII: Role of the Immune Metabolic Enzymes Indoleamine 2,3 Dyoxigenase-1 and Heme Oxygenase-1

**DOI:** 10.3389/fimmu.2020.00620

**Published:** 2020-04-15

**Authors:** Davide Matino, Sajjad Afraz, George Zhao, Paul Tieu, Marco Gargaro, Francesca Fallarino, Alfonso Iorio

**Affiliations:** ^1^Department of Medicine, McMaster University, Hamilton, ON, Canada; ^2^Thrombosis and Atherosclerosis Research Institute, Hamilton, ON, Canada; ^3^McMaster Faculty of Health Sciences, McMaster University, Hamilton, ON, Canada; ^4^Department of Experimental Medicine, University of Perugia, Perugia, Italy; ^5^Department of Health Research Methods, Evidence, and Impact, Hamilton, ON, Canada

**Keywords:** hemophilia, inhibitor, danger model, FVIII, IDO, HO-1, tolerance

## Abstract

The occurrence of neutralizing anti-FVIII antibodies is a major complication in the treatment of patients affected by hemophilia A. The immune response to FVIII is a complex, multi-factorial process that has been extensively studied for the past two decades. The reasons why only a proportion of hemophilic patients treated with FVIII concentrates develop a clinically significant immune response is incompletely understood. The “danger theory” has been proposed as a possible explanation to interpret the findings of some observational clinical studies highlighting the possible detrimental impact of inflammatory stimuli at the time of replacement therapy on inhibitor development. The host immune system is often challenged to react to FVIII under steady state or inflammatory conditions (e.g., bleeding, infections) although fine tuning of mechanisms of immune tolerance can control this reactivity and promote long-term unresponsiveness to the therapeutically administered factor. Recent studies have provided evidence that multiple interactions involving central and peripheral mechanisms of tolerance are integrated by the host immune system with the environmental conditions at the time of FVIII exposure and influence the balance between immunity and tolerance to FVIII. Here we review evidences showing the involvement of two key immunoregulatory oxygenase enzymes (IDO1, HO-1) that have been studied in hemophilia patients and pre-clinical models, showing that the ability of the host immune system to induce such regulatory proteins under inflammatory conditions can play important roles in the balance between immunity and tolerance to exogenous FVIII.

## Introduction

Hemophilia is a recessive X-linked inherited bleeding disorder caused by a deficient or defective protein needed for blood clotting. Hemophilia A (HA), characterized by Factor VIII (FVIII) deficiency is more common than hemophilia B (HB) (1), and is more often complicated by the occurrence of an immune response during treatment with the missing clotting factor (2). In particular, in patients affected by severe hemophilia (residual FVIII activity <1%) that require prophylactic administration of exogenous FVIII, the occurrence of neutralizing FVIII-specific IgG antibodies directed toward the infused clotting factor is frequent. In fact, up to 40% of treated patients will develop neutralizing antibodies (inhibitors). The development of inhibitors in hemophilia is a serious complication of factor replacement therapy. Immune tolerance to FVIII has been a major concern and interest of hematologists for many years, because the development of inhibitors significantly increases morbidity and lowers the quality of life within the hemophilia population (3). The reason why only a fraction of HA patients develop such an antibody response to FVIII has been a matter of debate among researchers. The last two decades have seen much progress toward the understanding of the basic science of inhibitor development but it is still not possible to predict which patients will develop an inhibitor on an individual basis. Multiple possible risk factors have been studied and have been categorized in two broad categories: patient-related (e.g., F8 gene mutation, family history of inhibitors, HLA haplotype, ethnicity, polymorphisms in immune genes), and environmental factors (e.g., intensity and type of treatment, type of FVIII product, age at first treatment, surgery, bleeding, vaccination) (4). Clinical studies dealing with patient-related risk factors have often reported conflicting results, except for the underlying F8 mutation, and in some instances adequate means of investigating these factors in the pre-clinical, basic science context were missing. Overall, decades of effort in investigating the immune response to FVIII have clearly highlighted the complexity of the process, which involves central and peripheral mechanisms of tolerance that are integrated by the host immune system with the environmental conditions at the time of FVIII exposure. Among the environmental factors, the role of the so-called “danger-signals” (e.g., vaccination, hemarthrosis, surgery) at the time of FVIII infusion in the development of inhibitors has attracted the interest of the scientific community, and offered a possible explanation for the intriguing question of why only a fraction of patients with severe hemophilia A develop an immune response to infused FVIII (5). In fact, the danger theory has been often indicated as a possible explanation for the observed phenomena and together with the self/non-self theory it has been used to conceptualize the development of inhibitors in hemophilia A. However, recently, it has become evident that mechanisms of peripheral tolerance in post-natal life are also important in the balance between tolerance and immunity to FVIII, and in particular the role of two key immunoregulatory enzymes, HO-1 and IDO1, has been described. The evidence from these pre-clinical and clinical studies also point to a possible different theoretical framework to interpret the data in the light of the combined role of central tolerance mechanisms during the early stages of T and B cells development, the danger theory and acquired mechanisms of peripheral tolerance at work throughout the adult life. In this review we will summarize the role of inducible peripheral tolerance mechanisms and the interplay between them and inflammatory/stress signals present in the environment.

## Self Recognition, Central Tolerance and Inhibitor Development in Hemophilia A: Is Central Tolerance Enough?

The immune response to FVIII is believed to develop as a classic CD4+ T cell-mediated response to an exogenous protein, where professional antigen presenting cells (APCs) internalize, process, and present FVIII-derived peptides to antigen-specific T cells (6). Activated T cells would then provide help to naïve FVIII-specific B cells that can ultimately differentiate either into memory B cells or antibody secreting cells that produce anti-FVIII antibodies (7). The reason why FVIII-reactive T and B cells exist can be explained by incomplete establishment of central tolerance, especially in cross-reactive material–negative (CRM-) patients. In these patients, FVIII-derived peptides could not be presented to T and B cells during their development in the primary lymphoid organs and the immune system of the patients have not been properly educated with FVIII during its ontogeny. Therefore, reactive cells are more likely to persist in the circulation. The association between certain F8 gene mutations and inhibitor development (8) highlights the importance of central tolerance mechanisms in controlling FVIII-reactive lymphocytes, suggesting the relevance of the long-standing idea that the immune system mainly distinguishes between self and non-self antigens. The self/non-self theory has been a pillar of immunology for many years and has helped to explain the development of tolerance or immune responses toward antigens in several contexts (9). In the case of hemophilia A, the relationship between the development of anti-FVIII antibodies and the type of F8 mutation was recognized more than 20 years ago. Mutations resulting in the absence (or severe truncation) of FVIII protein are associated with the highest risk of inhibitor formation, likely due to the prevention of a patient’s immune system from initiating early central tolerance to FVIII. Central tolerance refers to the regulatory mechanisms that occur at the early stages of B and T cell development in the bone marrow and thymus respectively, that culminates in the removal of strongly autoreactive B and T lymphocytes by clonal deletion, anergy, and receptor editing (10). T cells with low-affinity receptors to self antigens can also undergo a process of “clonal diversion” that promotes the differentiation of T regulatory cells. An interesting experimental proof of concept of central tolerance induction was developed by Madoiwa et al. (11). They demonstrated that the administration of FVIII into the thymus using a high-resolution ultrasound system results in the induction of FVIII-specific unresponsiveness in hemophilia A mice. The central tolerance process, however, is often imperfect and the escape of reactive cells into the periphery is still possible. FVIII reactive T cells can be found in both healthy donors and hemophilia A patients (12–15). Therefore, the recognition of self antigens (FVIII, in this case) is possible and can occur in healthy individuals. Consistent with these findings, anti-FVIII IgG antibodies can also be found in the general population as well as hemophilic patients with and without clinically relevant inhibitors (16–18). Additionally, only a fraction of patients with null mutations that most likely cannot undergo the physiological processes of central tolerance to FVIII will develop inhibitory antibodies. However, hemophilia A patients with less severe mutations that can still allow for a partial or complete production of FVIII antigen are still at risk of developing inhibitors. Altogether, these findings suggest that central tolerance is a first barrier against unwanted immune reactions against FVIII, but is not fool-proof and needs to be complemented with peripheral means of tolerance acquired during the adult life. Even though this has not been extensively studied in hemophilia yet, some evidence has been presented that mechanisms of peripheral tolerance are indeed associated with a negative inhibitor status in hemophilia A patients and can be exploited to control the immune response against exogenous FVIII. The next section of this review will describe the recent advances on how mechanisms of peripheral tolerance are involved in the control of the immune response to FVIII.

## The Role of Peripheral Tolerance Mechanisms in Hemophilia A

Peripheral tolerance develops after T and B cells mature and enter the peripheral tissues and lymph nodes (19). It is established by a number of partly overlapping mechanisms mostly involving control at the level of T cells, especially CD4+ helper T cells, which orchestrate immune responses and give B cells the confirmatory signals they need in order to produce antibodies. The critical pathway to provide the first T cells with information required to steer the immune response toward immunity or tolerance is mediated by peripheral APCs. During the primary immune response to FVIII, dendritic cells (DCs) are presumed to be the APCs primarily involved. However, DCs have a key role not only in promoting antigen-specific immunity, but also in acting as regulators of immune responses to antigens. Accumulating evidence indicates that indeed DCs can induce tolerance rather than immune activation to the antigen they present and a specific lack of peripheral DCs can lead to autoimmune pathology, demonstrating a role for DCs in peripheral tolerance (20, 21). The tolerogenic presentation of antigens by DCs can be promoted by anti-inflammatory enzymes. Most likely, congenital absence of FVIII prevents onset of central tolerance to FVIII, thus foisting effective control of FVIII-reactive lymphocytes on peripheral tolerance mechanisms at work in the post-natal life.

**FIGURE 1:**
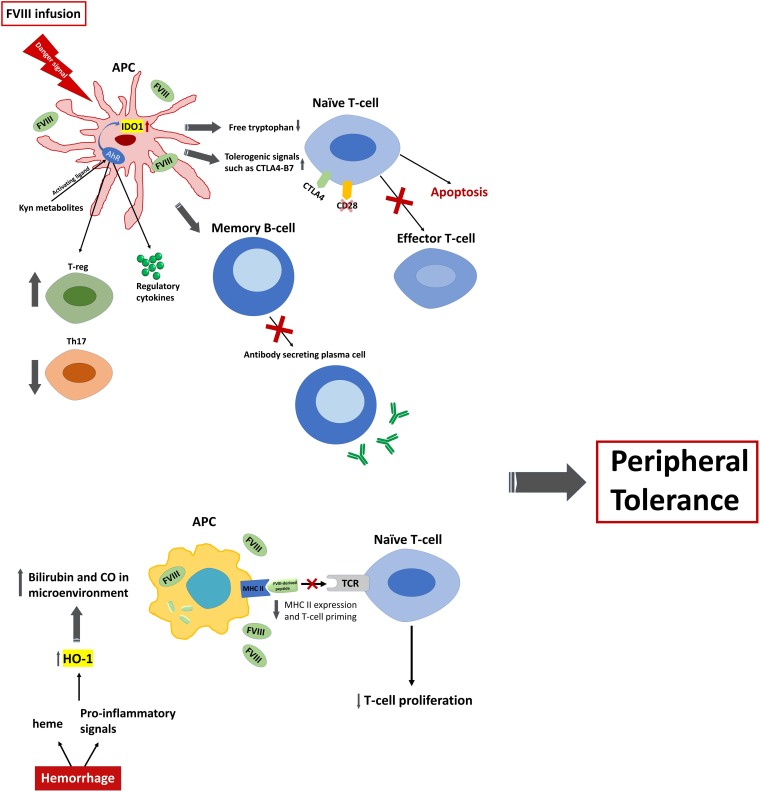
Schematic representation of tolerance induction via expression of IDO1 and HO-1 in hemophilia A. FVIII infusion in the presence of danger signal sensed by APCs, particularly DCs, can result in the expression of IDO1 by DCs. Expression of IDO1 at high levels in turn will influence several immune cells. IDO1 expression will arrest the proliferation of effector T cells and promote pro-apoptotic signals by depleting the microenvironment of Trp. It will also induce tolerogenic signals and anergy of naïve T cells by promoting the interaction of B7 ligand on the surface of APCs with CTLA4 receptor, rather than CD28, on the surface of naïve T cells. Additionally, accumulation of metabolites of kynurenine pathway, most importantly kynurenine and 3-HAA, can activate transcription factor AhR that leads to the upregulation of Tregs and downregulation of Th17. Moreover, AhR activation by these metabolites regulates the expression of TGF-β1 and IDO1 genes in DCs and will promote the expression of anti-inflammatory cytokines. In addition, inflammatory response via TLRs on DCs can inhibit the differentiation of FVIII specific memory B cells to antibody secreting cells through upregulation of IDO1 in the presence of high concentration of exogenous FVIII. On the other hand, presence of high concentration of heme, as well as inflammatory/stress signals which is caused by repeated episodes of bleeding in hemophilia A patients, can result in the generation of high levels of carbon monoxide (CO) and biliverdin in the microenvironment caused by increased activity of HO-1. This will decrease the MHC II expression that displays FVIII-derived peptides to TCR on naïve T cells. Consequently, fewer T cells will be primed and this will result in the reduction of T cell proliferation. As a consequence, activation of these two immune regulatory enzymes potentiate the induction of peripheral tolerance to FVIII and inhibit anti-FVIII inhibitor formation.

The potential role of regulators of peripheral tolerance has been recently explored in hemophilia, with a specific focus on two immunoregulatory enzymes: heme oxygenase-1 (HO-1) and indoleamine 2,3 dioxygenase (IDO; IDO-1). A schematic representation of HO-1 and IDO-1 effects is presented in Figure 1.

### Heme Oxygenase-1, an Enzyme With Oxidase Activity as Potential Regulator of Peripheral Tolerance to FVIII

#### Heme Oxygenase-1 in Immune Regulation

Heme oxygenase-1 (HO-1) is an enzyme that catabolizes the degradation of heme into ferrous ions, carbon monoxide (CO), and biliverdin (22). Biliverdin is further enzymatically reduced to bilirubin which possesses potent anti-inflammatory and anti-oxidant properties (23). HO-1 can be induced by the presence of heme as well as various stressors including proinflammatory cytokines and inflammatory stimuli (24). Thus, HO-1 induction exerts anti-inflammatory effects and when knocked-down in mice or deficient in humans, a chronic inflammatory phenotype is observed (25, 26). A growing body of literature has also shown that HO-1 is capable of inhibiting a variety of immune reactions (24). HO-1 upregulation has been shown *in vivo* to induce a protective effect against airway inflammation in allergic asthma and skin allergy models, potentially through the mechanism of enhancing expansion and suppression functions of CD4+/CD25+ Treg cells (27–29). In experimental autoimmune encephalomyelitis (EAE) models, a common animal model for multiple sclerosis, HO-1 knock-out mice develop severe EAE symptoms whereas mice with induced HO-1 exhibit reduced EAE symptoms (30).

Currently, the exact cellular mechanism of HO-1 induced immunosuppressive effects is still unclear. However, studies suggest that a large component may be attributed to the ability of HO-1 and the HO-1 catalyzed end products bilirubin and CO in inhibiting dendritic cell (DC) function (31–33). A recent study demonstrated that induction of HO-1 hinders DC maturation *in vitro* (31). This resulted in limited antigen presentation and activation of adaptive T cell responses as DCs after HO-1 induction exhibited diminished ability to stimulate proliferation of allogeneic CD4+ T cells (31). Other studies show that induction of HO-1 inhibited production of the pro-inflammatory cytokines IL-12, IL-6, TNF-a and type 1 interferons without inhibiting production of the anti-inflammatory cytokine IL-10 (32, 33). This cytokine environment may in turn promote expansion of Treg cells which has been seen in studies investigating the effect of HO-1 on allergic asthma (28). Although mechanisms need to be further elucidated, HO-1 evidently plays a role in regulating adaptive immune responses toward an anti-inflammatory phenotype.

#### HO-1 Induction Confers Tolerance to Exogenous FVIII in Experimental Hemophilia A Models

Interestingly, Dimitrov et al. demonstrated that HO-1 induction in FVIII-deficient mice prior to FVIII administration significantly reduces the anti-FVIII immune response (34). To induce HO-1 activity, mice were intravenously administered hemin, an oxidized form of heme. Results showed that out of the 9 mice that were administered hemin prior to treatment with FVIII, 8 were protected against inhibitor development and inhibitor levels only slightly above the lower limits of detection were found in the ninth mouse. On the other hand, animals that were given PBS instead of hemin developed high inhibiter titres after 3 weekly treatments (34). A similar trend was seen with anti-FVIII IgG levels. The involvement of HO-1 in the development of tolerance to exogenous FVIII was confirmed using pharmacological approaches. When the specific HO-1 inhibitor, SnMP, was co-administered with hemin prior to FVIII treatment, the protective effect of hemin alone was abrogated, and mice developed high levels of anti-FVIII IgG (34). SnMP was shown to not have an effect on anti-FVIII IgG development when administered alone (34). Additionally, when FVIII deficient mice were treated with CORM-3, a CO-releasing compound, or bilirubin instead of hemin, a diminished anti-FVIII IgG response similar to that when hemin was administered was observed (34). This suggests that the tolerogenic effect of HO-1 may be mainly attributed to the enzymatic pathway end products CO and bilirubin.

#### HO-1 May Exert Its Effects Through Modulation of Immune Cells

The protective effects of HO-1 may be due to modulation of immune cells that play an important role in FVIII antigen recognition, immune activation, and immune tolerance. Splenic macrophages are APCs critical in the primary immune response to exogenous FVIII and described as a location for exogenous FVIII accumulation due to antigen recognition and internalization (35, 36). Administration of hemin was associated with a significant decrease of major histocompatibility complex (MHC) class II expression on splenic macrophages as well as splenic dendritic cells, which play a similar antigen presenting role (34). Additionally, splenic T cells from HO-1 induced mice displayed decreased splenic T cell proliferation after injection with FVIII (34). However, no significant changes in T-regulatory cells were observed (34). These results taken together suggest that induction of HO-1 aids in the development of peripheral tolerance to exogenous FVIII in experimental hemophilia A, possibly due to diminishing capacity for antigen presentation and T-cell proliferation.

#### Increased HO-1 Expression Is Associated With Lesser Prevalence of Inhibitor Development in Humans

This relationship between HO-1 induction and tolerance to exogenous FVIII also translates clinically to hemophilia A patients. In humans, HO-1 is encoded by the HMOX1 gene and regulation of HO-1 expression is predominantly at the transcriptional level (37). Evidence suggests HO-1 expression is modulated by polymorphisms in the promoter region of the gene (37), whereby, the number of GT repeats in the promoter region of the HMOX1 gene is a determining factor of the capacity at which HO-1 is transcribed. Long GT repeats are associated with a diminished ability to express HO-1 in response to stimuli whereas shorter GT repeats result in greater HO-1 expression (38). Increased HO-1 induction in individuals with shorter GT repeats was associated with a lesser prevalence of inhibitor development. In a case-control study by Repesse et al. with a sample of 99 inhibitor-positive patients and 263 inhibitor-negative hemophilic patients, the number of GT repeats ranged between 14 and 38 repeats (37). After alleles for HMOX1 were divided into three subclasses depending on the number of GT repeats, where class S alleles contained <21 GT repeats, class M alleles contained 21–29 GT repeats, and class L alleles contained =30 GT repeats, results showed that hemophilic patients with the L/L genotype had a significantly greater prevalence of inhibitor development as compared to all other genotypes (37). Additionally, individuals with at least one L allele (L/L, L/M and L/S) were also significantly more likely to develop FVIII inhibitors compared to those that had no L allele (37). Even after controlling for hemophilia-causing mutations in a multivariable logistic regression, the authors found that this effect remained significant (37).

The results from these two studies strongly suggest that the induction of HO-1 exerts protective effects against anti-FVIII inhibitor formation. Additionally, certain individuals are genetically predisposed to greater HO-1 expression, thus making them more prone to inducing peripheral tolerance to FVIII through a HO-1 related mechanism.

### Indoleamine 2,3 Dioxygenase Another Enzyme With Oxidase Activity as Potential Regulator of Peripheral Tolerance to FVIII

#### IDO1 in Immune Regulation

Reciprocal interactions between metabolic pathways and immunity coordinate cross-talk between whole-body and immune cell functions and is involved in a variety of health and disease states (39). Among these pathways, metabolism of L-Tryptophan (Trp), one of the nine essential amino acid that is obtained exclusively from dietary intake in humans, regulates immune responses at multiple levels (40).

During homeostatic conditions in mammalian cells, 99% of dietary Trp is metabolized via the kynurenine pathway (41). Trp metabolism through the kynurenine pathway is catalyzed by three different enzymes namely, tryptophan 2,3-dioxygenase (TDO2), indoleamine 2,3 dioxygenase 1 (IDO1), and indoleamine 2,3 dioxygenase 2 (IDO2). Both TDO2 and IDO1 activities are rate limiting for Trp entry into the hepatic and extrahepatic kynurenine pathway, leading to Trp depletion and the production of a series of intercellular messengers molecules collectively known as kynurenines characterized by immunoregulatory, pro-apoptotic, and neuroactive properties (42–46). Both effects have been shown to be involved in the regulation of immune responses (47). Moreover, the IDO1 enzyme, as part of its moonlight activity, can also function as an intracellular signalling molecule whose posttranslational modifications are involved in the production of TGF-β by dendritic cells (DCs) (48, 49). All of these features in a combined fashion participate in the induction of immune regulatory pathways in various immune cells including T cells (50) and dendritic cells (48).

It is important to remember that IDO1 is an inducible enzyme whose expression can be strongly increased in immune cells by proinflammatory signals including Toll-like receptors (TLRs) and proinflammatory cytokines (45, 51–55).

In particular, co-culture of naïve CD4+ T cells with DCs expressing high level of IDO suppresses the proliferation of effector T cells and induced the expansion of Foxp3+ regulatory T cells *in vitro* (56–58). Several mechanisms have been attributed to immunosuppressive and immunoregulatory features of IDO1. IDO1 has very low Km for tryptophan, such that it readily depletes the microenvironment of tryptophan (59, 60). T cell proliferation is highly dependent on the presence of tryptophan. Therefore, IDO1 can alter T cell responses by locally depleting this essential amino acid and thus blocking the cell cycle in the interphase stage and arresting the proliferation of CD4+ T cells, which is key in the progression of humoral immune response (61, 62). There is also evidence for anergy of CD4+ T cells induced by accumulation of tryptophan catabolites as a result of IDO1 enzymatic activity (62, 63). The accumulation of downstream catabolites of kynurenine pathway have potent immunoregulatory effects and can induce the differentiation of naïve CD4+ T cells toward regulatory T cells (50, 57, 64). Among these, kynurenine and 3-Hydroxyanthranilic acid (3-HAA) were shown to induce Foxp3+ regulatory T cells (Tregs) expansion and inhibit non-Treg cell proliferation *in vitro* and *in vivo* similar to IDO1 (56). Kynurenine can promote similar effects by acting as activating ligands for transcription factor Aryl hydrocarbon receptor (AhR) leading to both regulation of systemic inflammatory response and increased ratio of Tregs to Th17 cells (49, 65). Moreover, a positive loop has been reported between Trp metabolism and AhR, as activation of AhR upregulates the expression of IDO1 in both mature and immature DCs (65, 66). Interestingly, the target for 3-HAA activity has recently been reported. 3-HAA was shown to activate the AhR coactivator, nuclear receptor coactivator 7 (NCOA) thus increasing the kynurenine induced effects (67).

Altogether these data suggest that IDO1 induction can control the host immune response and promote tolerance induction in several different contexts. Accordingly, dysregulation of Trp metabolism have been reported in various diseases including tumors, autoimmunity, and neurodegenerative diseases (40). Specifically, in different *in vivo* models, IDO1 expression has shown to have a protective effect on autoimmune encephalitis and pancreatic islet allograft (64, 68). In addition, IDO1 inhibition resulted in increased mortality and disease severity in an experimental model of T cell mediated colitis, and transfusion of IDO overexpressing DCs was associated with long term allograft survival of recipients in a mouse model of small bowel transplantation (56, 69). There are also numbers of studies that have reported the ability of tumor cells in evading host immune responses by expressing IDO1 (61, 70). All these evidences have recently stimulated interest in therapeutically targeting this pathway in various immune related disease conditions (40).

#### IDO1 and Allogenic Immune Response to FVIII in Hemophilia A

In hemophilia A, several immune cells are involved in directing the immune response toward inhibitor development, and antigen presentations by APCs and subsequent activation of FVIII-specific CD4+ T cells appear to play a key role (7, 71, 72).

The reduction of FVIII-specific CD4+ T cell activation, as well as amplification of regulatory subsets of T cells by IDO1 represents a potential mechanism for tolerance induction and a possible strategic means to restrain the anti-FVIII immune response in hemophilia A.

Correlation between IDO1 expression and anti-FVIII inhibitor development has been assessed in a few studies. In a study conducted by Liu et al., co-delivery of human FVIII and IDO1 genes into adult hemophilia A mice resulted in decreased anti-FVIII inhibitor development (73). In this study, expression of IDO1 protein significantly reduced anti-FVIII antibody levels, but did not completely inhibit the anti-FVIII immune responses. Here, high plasma level of kynurenine correlated with lower inhibitor level and apoptosis of T cells was observed in hemophilic mice that received IDO1 gene delivery. The author concluded that T cell apoptosis and blockade of T cell proliferation induced by IDO1 contributed to the modulation of the humoral immune response against FVIII in mice. In the same study, culture of murine peripheral blood mononuclear cells in the presence of kynurenine *in vitro* resulted in apoptosis of the cells (73).

In another study, high dose administration of TLR9 ligand (CpG-ODN) inhibited the differentiation of FVIII specific memory B cells to antibody secreting cells (ASCs) in the presence of high concentrations of FVIII in hemophilia A mice (74). Systemic high dose CpG-ODN was associated with increased expression of IDO by DCs in mouse models (74, 75). The author proposed that inhibitory effects of high concentrations of CpG-ODN on FVIII specific memory B cells may have been mediated by upregulated IDO1 expression by immune cells potentially involving DCs (74).

The IDO1 involvement in restraining FVIII antibody responses in hemophilia has been further confirmed by the study of Matino et al. where both IDO1 expression in hemophilic patients with or without inhibitor and the impact of IDO1 activity restraining FVIII alloantibodies in hemophilic mouse (i.e., F8 KO mice) were investigated (76). Specifically, in a cohort of 100 severe hemophilia A patients, the inhibitor-positive status was associated with dysfunctional activation of IDO1 in human CD11c+ APCs in response to the “environmental danger signal” CpG ODN acting as ligand for the Toll-like receptor 9 (TLR9) (76). In F8 KO mice, the animal model of hemophilia A, CpG-ODN administration and consequent induction of IDO1 in dendritic cells (DCs) was shown to prevent generation of anti-FVIII antibodies while promoting FVIII-specific FoxP3+ Tregs, which effects required both IDO1 and AhR in host immune cells (76).

Overall, all these studies could form a basis for further progress toward novel strategies involving Trp metabolism aimed at limiting FVIII alloantibody production and establishing tolerance to FVIII products. This could apply to patients at the beginning of prophylaxis to reduce the incidence rate of inhibitors, or in patients undergoing immune tolerance induction to increase the success in eradicating inhibitors to therapeutically administered FVIII protein.

## A New Integration of IDO1 and Potential HO-1 Activity in the Danger Model in Hemophilia A

A possibly unifying theoretical framework for the immune response to FVIII in HA patients has been sought after and the “danger theory” has been very well received among researchers in the field to explain, at least in part, the complex pattern of inhibitor development in hemophilia (77, 78).

The model, originally proposed by P. Matzinger, opposes the concept that the immune system’s primary goal is to discriminate between self and non-self (79). On the contrary, the danger theory proposes that the primary driving force of the host immune system is the need to detect and protect against danger. If a foreign or a self-antigen is not assessed as dangerous, tolerance should be the outcome. Therefore, according to this model the immune responses to FVIII could be influenced by the presence in the microenvironment of danger-signals. The immune system would discriminate not only on the basis of self vs. non-self but also by whether or not an antigen is perceived as dangerous. Theoretically, if FVIII is *per se* perceived as dangerous or if APCs somehow recognize tissue stress and injury at the time of FVIII exposure, they may present antigens to the immune system in that context. Potentially, this could happen when FVIII is administered during events such as hemarthrosis, surgery, trauma, vaccination, or infection. After administration, FVIII molecules can be captured and internalized by APCs, such as dendritic cells (DCs), and are processed and presented on the major histocompatibility (MHC) class II complex to naïve CD4 + T-cells. This process may occur in the presence of danger signals in the microenvironment. In fact, several concurrent events such as surgery or joint bleeds could theoretically result in tissue damage and the release in the extracellular milieu of damage-associated molecular patterns (DAMPs) (80). The presence of certain pathogen-derived molecules (pathogen-associated molecular patterns; PAMPs) could act in a similar way. Pattern recognition receptors (PRRs) on DCs surface can recognize and bind to DAMPs and to PAMPs (81). The binding to PRRs leads to the upregulation of essential costimulatory molecules (CD80/CD86) and other adhesion molecules, triggering the production of immune stimulatory cytokines. Activated T-cells can in turn activate FVIII-specific naive B-cells, which can expand and differentiate either into plasma cells, secreting anti-FVIII antibodies, or FVIII-specific B-memory cells.

In the absence of danger signals, DC maturation is not triggered by the engagement of pattern recognition receptors (PRR), co-stimulatory molecules on DCs are not upregulated, and this would prevent activation, clonal expansion, and acquisition of effector functions by T cells. The interaction between an APC not expressing co-stimulatory molecules would instead result in T-cells becoming anergic and not able to further stimulate B-cells.

This theoretical premise would support the effort to a) clearly identify the danger signals occurring during hemophilia A patients’ treatment and b) avoid such stimuli during FVIII administration.

However, clear evidence of direct and unequivocal effect on increasing immunogenicity of FVIII by danger signals is still missing.

### *In vitro* Studies

In the study by Pfistershammer et al., it was shown that neither FVIII, thrombin-activated FVIII, nor FVIII-VWF complex modulates the maturation of human dendritic cells (DCs) or their ability to stimulate T cells (82). Also, even though it has been hypothesized that FVIII could be immunogenic *in vivo* because of its procoagulant function, robust evidence in this sense is still lacking and conflicting results have been reported (83–85). However, human monocyte-derived DCs from healthy donors treated in combination with FVIII and a danger signal (LPS) at specific doses synergised in increasing DC activation, as characterised by increased expression of co-stimulatory molecules and secretion of pro-inflammatory cytokines (86). The results though would vary with the type of FVIII (recombinant vs plasma-derived) and the type and amount of co-applied danger signal. The authors concluded that also donor-intrinsic characteristics would play a relevant role.

### Joint Bleedings

A potential source of tissue damage and inflammation in hemophilic patients is recurrent joint bleed. This also requires treatment with FVIII and could then increase the risk of inhibitor development. In a hemophilia A mouse model of single knee puncture-induced haemarthrosis, the possible synergistic effect of joint bleeding on inhibitor development during FVIII therapy was investigated (87). The authors could not find an effect of joint bleeding on immune response to administered FVIII. On the other side, clinical studies reported conflicting results and could not show a consistent association between treatment of joint bleeding episodes and inhibitor development (88–90).

### Vaccinations

A possible influence of vaccinations at the time of FVIII administration has been also hypothesized and generated some discussion in the community of hemophilia treaters (91). In fact, in a similar way that the presence of adjuvants may stimulate the immune system, vaccinations might also act as a danger signal. The effect of influenza vaccinations given intramuscularly (i.m.) or intravenously (i.v.) prior to multiple infusions of FVIII was tested in a mouse model of hemophilia A (92). Surprisingly, the study found that vaccination did not increase the risk of inhibitor development and in fact resulted in reduced antibody responses to FVIII (92).

Clinical observational studies have not reported an increased risk of inhibitor with vaccinations (89, 90). More recently, a retrospective analysis evaluating the possible association between FVIII administration given in close proximity to vaccination and inhibitor development was conducted (93). A cohort of 375 previously untreated patients with severe haemophilia A was studied. The analysis was limited to patients receiving vaccinations between the first and 75th exposure day, when the risk of inhibitor development is highest. Interestingly, inhibitor developed in a similar but slightly lower frequency in patients receiving vaccinations with FVIII compared to patients receiving vaccinations without FVIII.

### Surgery

Surgery is another potential relevant immunological event in that it can create substantial tissue damage and be associated with release of endogenous DAMPs that could promote inhibitor development. A case-control study published in 2005 could not demonstrate a significant association between surgery and risk of inhibitor (89). In contrast, Gouw et al. combined individual patient data obtained from four recombinant FVIII product PUP studies performed between 1989 and 2001 (94). Peak treatment in correspondence of surgical procedures was associated with a 2.4 (CI 1.2–4.8) times increased risk of developing an anti-FVIII immune response. Similar results were obtained by Eckhardt et al. in a systematic review including four cohort studies and three case control studies. The analysis showed that intensive treatment at the time of surgery increased the risk of inhibitor development. The odds for inhibitor development in patients that received intensive treatment at the time of surgery was four times higher compared to patients that were treated for bleeding or prophylaxis (95). However, the association between surgery and inhibitor risk could not be confirmed in the a multicentre cohort study enrolling 606 previously untreated patients affected by severe hemophilia A (RODIN study) (96). Similarly, in a mouse model of hemophilia A, surgery did not increase inhibitor production (97). In this study, mice that underwent laparotomy were no more likely to develop anti-FVIII antibodies compared to those that did not. In addition, surgery did not result in higher-titre antibodies. However, surgery increased the production of inflammatory cytokines IL-1 and IL-6 and caused an upregulation of the expression of the costimulatory molecule CD80 on APCs.

In summary, some clinical studies have suggested a possible association of surgery with inhibitor formation, but results are not consistent and the large heterogeneity amongst included studies might also explain, at least in part, the differences. No pre-clinical model was able to prove a definite role for surgery in inhibitor development in hemophilia A mice so far.

### Avoiding Danger Signals During FVIII Exposure Cannot Prevent Inhibitor Development

Importantly, a possible consequence of the danger model is that administration of FVIII in the absence of danger signals, as is the case with prophylactic treatment, would promote tolerance to the deficient coagulation protein. In a pilot study, Kurnik et al. evaluated whether low-dose prophylaxis during the initial 20–50 EDs in combination with avoidance of immunological dangers signals could promote FVIII tolerance and reduce the incidence of inhibitors (98). In this prospective study consecutive patients were enrolled in 2 centers in Germany and an early prophylaxis regimen seemed to be associated with a significantly reduced risk of inhibitor development compared to patients treated with a standard prophylaxis regimen. This finding could not be replicated in a larger international prospective study (EPIC study; Early Prophylaxis Immunologic Challenge).

The study had to be terminated prematurely because of a higher than expected inhibitor incidence that seriously compromised the likelihood to reach the primary objective (8/19 patients, 42%) (99).

## An Integrated Model of Immune Response to FVIII

**FIGURE 2:**
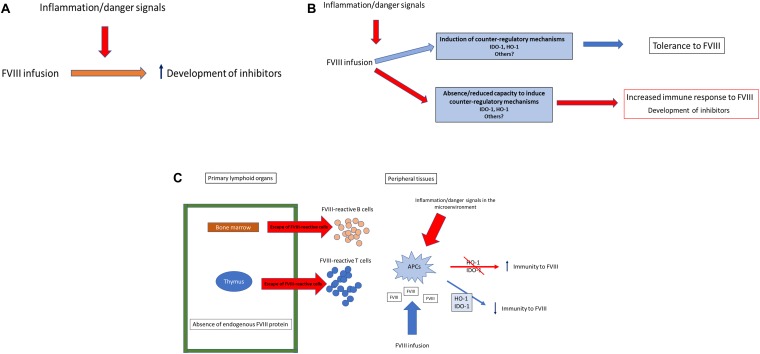
IDO and HO-1 in the immune response to FVIII. The presence of the danger model at the time of FVIII administration has been generally viewed as invariably increasing the risk for inhibitors development in all hemophilia A patients **(A)**. However, several studies have shown that there is a proportion of patients that cannot respond to inflammation/danger signals inducing counter-regulatory mechanisms of adaptive tolerance such as IDO and HO-1. The capacity of the host immune system to upregulate such mechanisms could tip the balance in favor of immunity or tolerance to FVIII **(B)**. Overall, in many severe hemophilia A patients that cannot produce any FVIII antigen, an altered central tolerance capacity is expected, resulting in an incomplete “barrier” preventing the escape of FVIII-reactive cells. However, in the FVIII-reactive cells can be controlled in the periphery by adaptive mechanisms of tolerance **(C)**.

Overall, a direct and univocal effect of inflammation/danger on inhibitor development in hemophilia A has not established yet, rather several of the aforementioned studies point to a substantial variability that is likely to be dependent on the host ability to control this external stimuli and how they are integrated in the immune response. It’s interesting to note that studies on both human and mice on IDO-1 and HO-1 indicate in fact that the response of the host to potential danger signals influences the outcome and directs the immune system toward tolerance or immunity. APCs, and in particular DCs, integrate complex environmental signals and have the capacity of directing the magnitude and polarity of the immune response. The influence of IDO-1 and HO-1 on APCs in promoting a tolerogenic response could represent both an important physiological control of the host response in this and other contexts, but also a possible target for a focused therapeutic manoeuvre. These peripheral mechanisms of tolerance are physiologically activated under stress conditions and can help regulate the response in an inflammatory micro-environment and the inability of the host to upregulate such systems could result in a more pronounced immune response, especially when reactive B and T cells are present in a significant amount because of an altered primary education of the immune system in the thymus and bone marrow. This could indeed be the case in hemophilia A patients, particularly for those that have mutations preventing the production of FVIII antigen (cross-reactive material negative; CRM-). In these patients, control of the FVIII immune response is more heavily dependent on the capacity of counteractive activity of immunoregulatory pathways of peripheral tolerance such as IDO-1 and HO-1. The results of the published studies clearly point toward a variability in the induction of such enzymes and failure to activate such regulatory mechanisms are associated to inhibitor development in HA patients and increased inhibitor production in experimental models. Taken together, results of clinical and pre-clinical studies would suggest that even though danger signals are often present at the time of FVIII administration (e.g., hemarthrosis), this does not always determine the occurrence of an immunogenic response to FVIII but can also allow the development of tolerance through the upregulation of systems of adaptive immunity that actively promote tolerance and control inflammation (Figure 2). This is in fact the most common outcome of the encounter between exogenous FVIII and the patient’s immune system and suggests that FVIII is indeed constantly assessed by the host immune system and actively tolerated in a substantial proportion of patients (71). Monitoring the function and capacity of inducible mechanisms of peripheral tolerance such as IDO-1 and HO-1 in hemophilic patients receiving replacement therapy might lead to a better understanding of the process that result in tolerance or immunity to clotting factor concentrates and contribute to the generation of focused and strategic intervention to promote favorable immunological outcomes in this challenging context.

## Author Contributions

DM and AI participated in the planning and writing, reviewed and edited the manuscript, conceptualized the figures. SA, GZ, and PT wrote parts of the article and generated the figures. FF and MG wrote parts of the manuscript.

## Conflict of Interest

The authors declare that the research was conducted in the absence of any commercial or financial relationships that could be construed as a potential conflict of interest.

## Funidng

This work was supported by the Italian Association for Cancer Research (AIRC 19903 to FF) and Telethon (GGP17094 to FF), and the research projects of National Interest (Prin 2017BZEREZ to FF).

## References

[1] Stonebraker J Chambost H Makris M Coffin D Herr C Germini F Establishing the prevalence and prevalence at birth of hemophilia in males: a meta-analytic approach using national registries. *Ann Intern Med*. (2019) 171:540–6. 10.7326/M19-1208 31499529

[2] Castaman G Matino D. Hemophilia A and B: molecular and clinical characteristics of similar, but different diseases. *Haematologica*. (2019) 104:1702–9. 31399527 10.3324/haematol.2019.221093 PMC6717582

[3] Walsh CE Jiménez-Yuste V Auerswald G Grancha S. The burden of inhibitors in haemophilia patients. *Thromb Haemost*. (2016) 116:S10–7. 10.1160/TH16-01-0049 27528280

[4] Peyvandi F Garagiola I. Product type and other environmental risk factors for inhibitor development in severe hemophilia A. *Res Pract Thromb Haemost*. (2018) 2:220–7. 30046724 10.1002/rth2.12094 PMC6055565

[5] Lövgren KM Søndergaard H Skov S Wiinberg B. Non−genetic risk factors in haemophilia A inhibitor management–the danger theory and the use of animal models. *Haemophilia*. (2016) 22:657–66. 10.1111/hae.13075 27562315

[6] Lacroix-Desmazes S Navarrete AM André S Bayry J Kaveri SV Dasgupta S. Dynamics of factor VIII interactions determine its immunologic fate in hemophilia a. *Blood*. (2008) 112:240–9. 10.1182/blood-2008-02-124941 18469198

[7] Delignat S Rayes J Russick J Kaveri SV Lacroix-Desmazes S. Inhibitor formation in congenital hemophilia A: an immunological perspective. *Semin Thromb Hemost*. (2018) 44:517–30. 10.1055/s-0038-1657777 29864775

[8] Gouw SC van den Berg HM Oldenburg J Astermark J de Groot PG Margaglione M F8 gene mutation type and inhibitor development in patients with severe hemophilia A: systematic review and meta-analysis. *Blood*. (2012) 119:2922–34. 10.1182/blood-2011-09-379453 22282501

[9] Medzhitov R Janeway CA Jr. How does the immune system distinguish self from nonself? *Sem Immunol*. (2000) 12:185–8. 10.1006/smim.2000.0230 10910738

[10] Xing Y Hogquist KAT-. cell tolerance: central and peripheral. *Cold Spring Harb Perspect Biol*. (2012) 4:a006957. 10.1101/cshperspect.a006957 22661634 PMC3367546

[11] Madoiwa S Yamauchi T Kobayashi E Hakamata Y Dokai M Makino N Induction of factor VIII−specific unresponsiveness by intrathymic factor VIII injection in murine hemophilia A. *J Thromb Haemost*. (2009) 7:811–24. 10.1111/j.1538-7836.2009.03314.x 19220731

[12] Reding MT Wu H Krampf M Okita DK Diethelm-Okita BM Christie BA Sensitization of CD4+ T cells to coagulation factor VIII: response in congenital and acquired hemophilia patients and in healthy subjects. *Thromb Haemost*. (2000) 84:643–52. 11057864

[13] Hu G Okita DK Diethelm−Okita BM Conti−Fine BM. Recognition of coagulation factor VIII by CD4+ T cells of healthy humans. *J Thromb Haemost*. (2003) 1:2159–66. 14521599 10.1046/j.1538-7836.2003.00366.x

[14] Meunier S Menier C Marcon E Lacroix-Desmazes S Maillère B. CD4 T cells specific for factor VIII are present at high frequency in healthy donors and comprise naïve and memory cells. *Blood Adv*. (2017) 1:1842–7. 29296830 10.1182/bloodadvances.2017008706 PMC5728100

[15] Jacquemin M Vantomme V Buhot C Lavend’homme R Burny W Demotte N CD4+ T-cell clones specific for wild-type factor VIII: a molecular mechanism responsible for a higher incidence of inhibitor formation in mild/moderate hemophilia A. *Blood*. (2003) 101:1351–8. 12393451 10.1182/blood-2002-05-1369

[16] Whelan SFJ Hofbauer CJ Horling FM Allacher P Wolfsegger MJ Oldenburg J Distinct characteristics of antibody responses against factor VIII in healthy individuals and in different cohorts of hemophilia A patients. *Blood*. (2013) 121:1039–48. 10.1182/blood-2012-07-444877 23243272

[17] Hofbauer CJ Whelan SFJ Hirschler M Allacher P Horling FM Lawo J-P Affinity of FVIII-specific antibodies reveals major differences between neutralizing and nonneutralizing antibodies in humans. *Blood*. (2015) 125:1180–8. 10.1182/blood-2014-09-598268 25515962

[18] Moreau A Lacroix-Desmazes S Stieltjes N Saenko E Kaveri SV D’Oiron R Antibodies to the FVIII light chain that neutralize FVIII procoagulant activity are present in plasma of nonresponder patients with severe hemophilia A and in normal polyclonal human IgG. *Blood*. (2000) 95:3435–41. 10828026

[19] Mathis D Benoist C. Back to central tolerance. *Immunity*. (2004) 20:509–16. 15142520 10.1016/s1074-7613(04)00111-6

[20] Mueller DL. Mechanisms maintaining peripheral tolerance. *Nat Immunol*. (2010) 11:21. 10.1038/ni.1817 20016506

[21] Ohnmacht C Pullner A King SBS Drexler I Meier S Brocker T Constitutive ablation of dendritic cells breaks self-tolerance of CD4 T cells and results in spontaneous fatal autoimmunity. *J Exp Med*. (2009) 206:549–59. 10.1084/jem.20082394 19237601 PMC2699126

[22] Schuller DJ Wilks A de Montellano PRO Poulos TL. Crystal structure of human heme oxygenase-1. *Nat Struct Mol Biol*. (1999) 6:860. 10.1038/12319 10467099

[23] Abraham NG Kappas A. Pharmacological and clinical aspects of heme oxygenase. *Pharmacol Rev*. (2008) 60:79–127. 10.1124/pr.107.07104 18323402

[24] Blancou P Tardif V Simon T Rémy S Carreño L Kalergis A Immunoregulatory properties of heme oxygenase-1. In: Cuturi M Anegon I editors. *Suppression and Regulation of Immune Responses. Methods in Molecular Biology (Methods and Protocols)* (Vol. 677), Totowa, NJ: Humana Press (2010). 10.1007/978-1-60761-869-0_18 20941616

[25] Kapturczak MH Wasserfall C Brusko T Campbell-Thompson M Ellis TM Atkinson MA Heme oxygenase-1 modulates early inflammatory responses. *Am J Pathol*. (2004) 165(3):1045–53. 15331427 10.1016/S0002-9440(10)63365-2 PMC1618611

[26] Yachie A Niida Y Wada T Igarashi N Kaneda H Toma T Oxidative stress causes enhanced endothelial cell injury in human heme oxygenase-1 deficiency. *J Clin Invest*. (1999) 103(1):129–35. 9884342 10.1172/JCI4165 PMC407858

[27] Xia Z-W Xu L-Q Zhong W-W Wei J-J Li N-L Shao J Heme oxygenase-1 attenuates ovalbumin-induced airway inflammation by up-regulation of foxp3 T-regulatory cells, interleukin-10, and membrane-bound transforming growth factor- 1. *Am J Pathol*. (2007) 171(6):1904–14. 17991714 10.2353/ajpath.2007.070096 PMC2111113

[28] Xia Z-W Zhong W-W Xu L-Q Sun J-L Shen Q-X Wang J-G Heme oxygenase-1-mediated CD4+CD25high regulatory T cells suppress allergic airway inflammation. *J Immunol*. (2006) 177(9):5936–45. 17056518 10.4049/jimmunol.177.9.5936

[29] Listopad J Asadullah K Sievers C Ritter T Meisel C Sabat R Heme oxygenase-1 inhibits T cell-dependent skin inflammation and differentiation and function of antigen-presenting cells. *Exp Dermatol*. (2007) 16(8):661–70. 17620093 10.1111/j.1600-0625.2007.00581.x

[30] Chora AA Fontoura P Cunha A Pais TF Cardoso S Ho PP Heme oxygenase-1 and carbon monoxide suppress autoimmune neuroinflammation. *J Clin Invest*. (2007) 117(2):438–47. 17256058 10.1172/JCI28844 PMC1770945

[31] Campbell NK Fitzgerald HK Malara A Hambly R Sweeney CM Kirby B Naturally derived Heme-Oxygenase 1 inducers attenuate inflammatory responses in human dendritic cells and T cells: relevance for psoriasis treatment. *Sci Rep*. (2018) 8:1–15. 10.1038/s41598-018-28488-6 29980703 PMC6035209

[32] Chauveau C Rémy S Royer PJ Hill M Tanguy-Royer S Hubert F-X Heme oxygenase-1 expression inhibits dendritic cell maturation and proinflammatory function but conserves IL-10 expression. *Blood*. (2005) 106(5):1694–702. 15920011 10.1182/blood-2005-02-0494

[33] Rémy S Blancou P Tesson L Tardif V Brion R Royer PJ Carbon monoxide inhibits TLR-induced dendritic cell immunogenicity. *J Immunol*. (2009) 182(4):1877–84. 10.4049/jimmunol.0802436 19201840

[34] Dimitrov JD Dasgupta S Navarrete A-M Delignat S Repesse Y Meslier Y Induction of heme oxygenase-1 in factor VIII–deficient mice reduces the immune response to therapeutic factor VIII. *Blood*. (2010) 115:2682–5. 10.1182/blood-2009-04-216408 19890094

[35] Navarrete A Dasgupta S Delignat S Caligiuri G Christophe OD Bayry J Splenic marginal zone antigen−presenting cells are critical for the primary allo−immune response to therapeutic factor VIII in hemophilia A. *J Thromb Haemost*. (2009) 7:1816–23. 10.1111/j.1538-7836.2009.03571.x 19682235

[36] van Schooten CJ Shahbazi S Groot E Oortwijn BD van den Berg HM Denis CV Macrophages contribute to the cellular uptake of von Willebrand factor and factor VIII in vivo. *Blood*. (2008) 112:1704–12. 10.1182/blood-2008-01-133181 18559674

[37] Repessé Y Peyron I Dimitrov JD Dasgupta S Moshai EF Costa C Development of inhibitory antibodies to therapeutic factor VIII in severe hemophilia A is associated with microsatellite polymorphisms in the HMOX1 promoter. *Haematologica*. (2013) 98:1650–5. 10.3324/haematol.2013.084665 23716558 PMC3789472

[38] Brydun A Watari Y Yamamoto Y Okuhara K Teragawa H Kono F Reduced expression of heme oxygenase-1 in patients with coronary atherosclerosis. *Hypertens Res*. (2007) 30:341. 10.1291/hypres.30.341 17541213

[39] Pearce EJ Pearce EL. Immunometabolism in 2017: driving immunity: all roads lead to metabolism. *Nat Rev Immunol*. (2017) 18:81. 10.1038/nri.2017.139 29226911 PMC6312922

[40] Platten M Nollen EAA Röhrig UF Fallarino F Opitz CA. Tryptophan metabolism as a common therapeutic target in cancer, neurodegeneration and beyond. *Nat Rev Drug Discov*. (2019) 18:379–401. 10.1038/s41573-019-0016-5 30760888

[41] Russo S Kema IP Fokkema RM Boon JC Willemse PHB de Vries EGE Tryptophan as a link between psychopathology and somatic states. *Psychosom Med*. (2003) 65:665–71. 12883120 10.1097/01.psy.0000078188.74020.cc

[42] Grohmann U Fallarino F Puccetti P. Tolerance, DCs and tryptophan: much ado about IDO. *Trends Immunol*. (2003) 24:242–8. 12738417 10.1016/s1471-4906(03)00072-3

[43] Mellor AL Munn DH. IDO expression by dendritic cells: tolerance and tryptophan catabolism. *Nat Rev Immunol*. (2004) 4:762. 10.1038/nri1457 15459668

[44] Kolodziej LR Paleolog EM Williams RO. Kynurenine metabolism in health and disease. *Amino Acids*. (2011) 41:1173–83. 10.1007/s00726-010-0787-9 20972599

[45] Fallarino F Grohmann U Hwang KW Orabona C Vacca C Bianchi R Modulation of tryptophan catabolism by regulatory T cells. *Nat Immunol*. (2003) 4:1206. 10.1038/ni1003 14578884

[46] Romani L Fallarino F De Luca A Montagnoli C D’Angelo C Zelante T Defective tryptophan catabolism underlies inflammation in mouse chronic granulomatous disease. *Nature*. (2008) 451:211–5. 10.1038/nature06471 18185592

[47] Grohmann U Mondanelli G Belladonna ML Orabona C Pallotta MT Iacono A Amino-acid sensing and degrading pathways in immune regulation. *Cytokine Growth Factor Rev*. (2017) 35:37–45. 10.1016/j.cytogfr.2017.05.004 28545736

[48] Pallotta MT Orabona C Volpi C Vacca C Belladonna ML Bianchi R Indoleamine 2, 3-dioxygenase is a signaling protein in long-term tolerance by dendritic cells. *Nat Immunol*. (2011) 12:870. 10.1038/ni.2077 21804557

[49] Bessede A Gargaro M Pallotta MT Matino D Servillo G Brunacci C Aryl hydrocarbon receptor control of a disease tolerance defence pathway. *Nature*. (2014) 511:184–90. 10.1038/nature13323 24930766 PMC4098076

[50] Fallarino F Grohmann U You S McGrath BC Cavener DR Vacca C The combined effects of tryptophan starvation and tryptophan catabolites down-regulate T cell receptor ζ-chain and induce a regulatory phenotype in naive T cells. *J Immunol*. (2006) 176:6752–61. 10.4049/jimmunol.176.11.6752 16709834

[51] Agaugué S Perrin-Cocon L Coutant F André P Lotteau V. 1-Methyl-tryptophan can interfere with TLR signaling in dendritic cells independently of IDO activity. *J Immunol*. (2006) 177:2061–71. 16887964 10.4049/jimmunol.177.4.2061 PMC2377404

[52] Popov A Schultze JL. IDO-. expressing regulatory dendritic cells in cancer and chronic infection. *J Mol Med*. (2008) 86:145–60. 17876564 10.1007/s00109-007-0262-6

[53] Volpi C Fallarino F Pallotta MT Bianchi R Vacca C Belladonna ML High doses of CpG oligodeoxynucleotides stimulate a tolerogenic TLR9–TRIF pathway. *Nat Commun*. (2013) 4:1852. 10.1038/ncomms2874 23673637

[54] Puccetti P. On watching the watchers: IDO and type I/II IFN. *Eur J Immunol*. (2007) 37:876–9. 17393386 10.1002/eji.200737184

[55] Grohmann U Volpi C Fallarino F Bozza S Bianchi R Vacca C Reverse signaling through GITR ligand enables dexamethasone to activate IDO in allergy. *Nat Med*. (2007) 13:579. 10.1038/nm1563 17417651

[56] Xie FT Cao J Sen Zhao J Yu Y Qi F Dai XC. IDO expressing dendritic cells suppress allograft rejection of small bowel transplantation in mice by expansion of Foxp3 + regulatory T cells. *Transpl Immunol*. (2015) 33:69–77. 10.1016/j.trim.2015.05.003 26002283

[57] Chen W Liang X Peterson AJ Munn DH Bruce BR. The indoleamine 2,3-dioxygenase pathway is essential for human plasmacytoid dendritic cell-induced adaptive T regulatory cell generation. *Blood*. (2007) 110:1344–1344. 10.1182/blood.v110.11.1344.1344 18832696 PMC2614675

[58] Baren Van N Pilotte L Moulin P Larrieu P Gutierrez-roelens I Renauld J Extensive pro fi ling of the expression of the indoleamine 2, 3-dioxygenase 1 protein in normal and tumoral human tissues. *Cancer Immunol Res*. (2015) 3:161–73. 10.1158/2326-6066.CIR-14-0137 25271151

[59] Basran J Booth ES Lee M Handa S Raven EL. Analysis of reaction intermediates in tryptophan 2,3-dioxygenase: a comparison with indoleamine 2,3-dioxygenase. *Biochemistry*. (2016) 55:6743–50. 10.1021/acs.biochem.6b01005 27951658

[60] Ball HJ Mowat CG. Human indoleamine 2, 3-dioxygenase-2 has substrate specificity and inhibition characteristics distinct from those of indoleamine. *Amino Acids*. (2014) 46:2155–63. 10.1007/s00726-014-1766-3 24875753

[61] Uyttenhove C Pilotte L Théate I Stroobant V Colau D Parmentier N Evidence for a tumoral immune resistance mechanism based on tryptophan degradation by indoleamine 2,3-dioxygenase. *Nat Med*. (2003) 9:1269–74. 10.1038/nm934 14502282

[62] Terness P Bauer TM Röse L Dufter C Watzlik A Simon H Inhibition of allogeneic T cell proliferation by indoleamine 2,3-dioxygenase-expressing dendritic cells: mediation of suppression by tryptophan metabolites. *J Exp Med*. (2002) 196:447–57. 10.1084/jem.20020052 12186837 PMC2196057

[63] Munn DH Sharma MD Baban B Harding HP Zhang Y Ron D GCN2 kinase in T cells mediates proliferative arrest and anergy induction in response to indoleamine 2,3-dioxygenase. *Immunity*. (2005) 22:633–42. 10.1016/j.immuni.2005.03.013 15894280

[64] Yan Y Zhang G-X Gran B Fallarino F Yu S Li H Upregulates regulatory t cells via tryptophan catabolite and suppresses encephalitogenic T cell responses in experimental autoimmune encephalomyelitis. *J Immunol*. (2010) 185:5953–61. 10.4049/jimmunol.1001628 20944000 PMC2998795

[65] Nguyen NT Kimura A Nakahama T Chinen I Masuda K Nohara K Aryl hydrocarbon receptor negatively regulates dendritic cell immunogenicity via a kynurenine-dependent mechanism. *Proc Natl Acad Sci USA*. (2010) 107:19961–6. 10.1073/pnas.1014465107 21041655 PMC2993339

[66] Mezrich JD Fechner JH Zhang X Johnson BP Burlingham WJ Bradfield CA. An interaction between kynurenine and the Aryl hydrocarbon receptor can generate regulatory T cells. *J Immunol*. (2010) 185:3190–8. 10.4049/jimmunol.0903670 20720200 PMC2952546

[67] Gargaro M Vacca C Massari S Scalisi G Manni G Mondanelli G Engagement of nuclear coactivator 7 by 3-hydroxyanthranilic acid enhances activation of aryl hydrocarbon receptor in immunoregulatory dendritic cells. *Front Immunol*. (2019) 10:1–14. 10.3389/fimmu.2019.01973 31481962 PMC6710348

[68] Alexander AM Crawford M Bertera S Rudert WA Takikawa O Robbins PD Indoleamine 2,3-dioxygenase expression in transplanted NOD islets prolongs graft survival after adoptive transfer of diabetogenic splenocytes. *Diabetes*. (2002) 51:356–65. 11812742 10.2337/diabetes.51.2.356

[69] Gurtner GJ Newberry RD Schloemann SR McDonald KG Stenson WF. Inhibition of indoleamine 2,3-dioxygenase augments trinitrobenzene sulfonic acid colitis in mice. *Gastroenterology*. (2003) 125:1762–73. 10.1053/j.gastro.2003.08.031 14724829

[70] van Baren N Van den Eynde BJ. Tryptophan-degrading enzymes in tumoral immune resistance. *Front Immunol*. (2015) 6:34. 10.3389/fimmu.2015.00034 25691885 PMC4315104

[71] Varthaman A Lacroix-Desmazes S. Pathogenic immune response to therapeutic factor VIII: Exacerbated response or failed induction of tolerance? *Haematologica*. (2019) 104:236–44. 10.3324/haematol.2018.206383 30514798 PMC6355482

[72] Lacroix-Desmazes S Scott DW Goudemand J Van Den Berg M Makris M Van Velzen AS Summary report of the first international conference on inhibitors in haemophilia A. *Blood Transfus*. (2017) 15:568–76. 10.2450/2016.0252-16 27893354 PMC5649967

[73] Liu L Liu H Mah C Fletcher BS. Indoleamine 2,3-dioxygenase attenuates inhibitor development in gene-therapy-treated hemophilia A mice. *Gene Ther*. (2009) 16:724–33. 10.1038/gt.2009.13 19262614

[74] Allacher P Baumgartner CK Pordes AG Ahmad RU Schwarz HP Reipert BM. Stimulation and inhibition of FVIII-specific memory B-cell responses by CpG-B (ODN 1826), a ligand for Toll-like receptor 9. *Blood*. (2011) 117:259–67. 10.1182/blood-2010-06-289009 20889922

[75] Puccetti P Grohmann U. IDO and regulatory T cells: a role for reverse signalling and non-canonical NF-κB activation. *Nat Rev Immunol*. (2007) 7:817–23. 10.1038/nri2163 17767193

[76] Matino D Gargaro M Santagostino E Di Minno MND Castaman G Morfini M IDO1 suppresses inhibitor development in hemophilia A treated with factor VIII. *J Clin Invest*. (2015) 125:3766–81. 10.1172/JCI81859 26426076 PMC4607121

[77] Wroblewska A Reipert BM Pratt KP Voorberg J. Dangerous liaisons: how the immune system deals with factor VIII. *J Thromb Haemost*. (2013) 11:47–55. 10.1111/jth.12065 23140211

[78] Astermark J Altisent C Batorova A Diniz MJ Gringeri A Holme PA Non−genetic risk factors and the development of inhibitors in haemophilia: a comprehensive review and consensus report. *Haemophilia*. (2010) 16:747–66. 20398077 10.1111/j.1365-2516.2010.02231.x

[79] Matzinger P. Tolerance, danger, and the extended family. *Annu Rev Immunol*. (1994) 12:991–1045. 10.1146/annurev.iy.12.040194.005015 8011301

[80] Van Helden PMW Van Haren SD Fijnvandraat K Marijke van den Berg H Voorberg J. Factor VIII−specific B cell responses in haemophilia A patients with inhibitors. *Haemophilia*. (2010) 16:35–43. 10.1111/j.1365-2516.2010.02215.x 20536984

[81] Bianchi ME. DAMPs, PAMPs and alarmins: all we need to know about danger. *J Leukoc Biol*. (2007) 81:1–5. 10.1189/jlb.0306164 17032697

[82] Pfistershammer K Stöckl J Siekmann J Turecek PL Schwarz HP Reipert BM. Recombinant factor VIII and factor VIII-von Willebrand factor complex do not present danger signals for human dendritic cells. *Thromb Haemost*. (2006) 96:309–16. 16953272 10.1160/TH05-11-0729

[83] Skupsky J Zhang A-H Su Y Scott DW. A. role for thrombin in the initiation of the immune response to therapeutic factor VIII. *Blood*. (2009) 114:4741–8. 10.1182/blood-2008-10-186452 19794141 PMC2780310

[84] Meeks SL Cox CL Healey JF Parker ET Doshi BS Gangadharan B Major determinant of the immunogenicity of factor VIII in a murine model is independent of its procoagulant function. *Blood*. (2012) 120:2512–20. 22855607 10.1182/blood-2012-02-412361 PMC3448263

[85] Gangadharan B Delignat S Ollivier V Gupta N Mackman N Kaveri SV Role of coagulation−associated processes on factor VIII immunogenicity in a mouse model of severe hemophilia A. *J Thromb Haemost*. (2014) 12:2065–9. 25267332 10.1111/jth.12740

[86] Miller L Weissmüller S Ringler E Crauwels P van Zandbergen G Seitz R Danger signal-dependent activation of human dendritic cells by plasma-derived factor VIII products. *Thromb Haemost*. (2015) 114:268–76. 10.1160/TH14-09-0789 25947149

[87] Peyron I Dimitrov JD Delignat S Gangadharan B Planchais C Kaveri SV Haemarthrosis and arthropathy do not favour the development of factor VIII inhibitors in severe haemophilia A mice. *Haemoph Off J World Fed Hemoph*. (2015) 21:e94. 10.1111/hae.12579 25471131

[88] Gouw SC van der Bom JG Van Den Berg HM. Treatment-related risk factors of inhibitor development in previously untreated patients with hemophilia A: the CANAL cohort study. *Blood*. (2007) 109:4648–54. 17289808 10.1182/blood-2006-11-056291

[89] Santagostino E Mancuso ME Rocino A Mancuso G Mazzucconi MG Tagliaferri A Environmental risk factors for inhibitor development in children with haemophilia A: a case–control study. *Br J Haematol*. (2005) 130:422–7. 16042693 10.1111/j.1365-2141.2005.05605.x

[90] Maclean PS Richards M Williams M Collins P Liesner R Keeling DM Treatment related factors and inhibitor development in children with severe haemophilia A. *Haemophilia*. (2011) 17:282–7. 10.1111/j.1365-2516.2010.02422.x 21070501

[91] Santagostino E Riva A Cesaro S Esposito S Matino D Mazzucchelli RI Consensus statements on vaccination in patients with haemophilia—Results from the Italian haemophilia and vaccinations (HEVA) project. *Haemophilia*. (2019) 25:656–67. 30990961 10.1111/hae.13756 PMC6850056

[92] Lai JD Moorehead PC Sponagle K Steinitz KN Reipert BM Hough C Concurrent influenza vaccination reduces anti-FVIII antibody responses in murine hemophilia A. *Blood*. (2016) 127:3439–49. 10.1182/blood-2015-11-679282 27034428

[93] Platokouki H Fischer K Gouw SC Rafowicz A Carcao M Kenet G Vaccinations are not associated with inhibitor development in boys with severe haemophilia A. *Haemophilia*. (2018) 24:283–90. 10.1111/hae.13387 29243367

[94] Gouw SC Van Den Berg HM Le Cessie S Van Der Bom JG. Treatment characteristics and the risk of inhibitor development: a multicenter cohort study among previously untreated patients with severe hemophilia A. *J Thromb Haemost*. (2007) 5:1383–90. 17456190 10.1111/j.1538-7836.2007.02595.x

[95] Eckhardt CL Van der Bom JG Van der Naald M Peters M Kamphuisen PW Fijnvandraat K. Surgery and inhibitor development in hemophilia A: a systematic review. *J Thromb Haemost*. (2011) 9:1948–58. 10.1111/j.1538-7836.2011.04467.x 21838755

[96] Gouw SC van den Berg HM Fischer K Auerswald G Carcao M Chalmers E Intensity of factor VIII treatment and inhibitor development in children with severe hemophilia A: the RODIN study. *Blood*. (2013) 121:4046–55. 10.1182/blood-2012-09-457036 23553768

[97] Moorehead PC Waters B Sponagle K Steinitz KN Reipert BM Lillicrap D. Surgical injury alone does not provoke the development of factor VIII inhibitors in mouse models of hemophilia A. *Blood*. (2012) 120:627 10.1182/blood.v120.21.627.627

[98] Kurnik K Bidlingmaier C Engl W Chehadeh H Reipert B Auerswald G. New early prophylaxis regimen that avoids immunological danger signals can reduce FVIII inhibitor development. *Haemophilia*. (2010) 16:256–62. 10.1111/j.1365-2516.2009.02122.x 19878331

[99] Auerswald G Kurnik K Aledort LM Chehadeh H Loew−Baselli A Steinitz K The EPIC study: a lesson to learn. *Haemophilia*. (2015) 21:622–8. 10.1111/hae.12666 25912619

